# Click Reactions in Kinetic Target-Guided Synthesis: Progress in the Discovery of Inhibitors for Biological Targets

**DOI:** 10.3390/mps9020054

**Published:** 2026-04-01

**Authors:** Prakash T. Parvatkar, Nishikant Satam

**Affiliations:** 1 Department of Chemistry and Chemical Biology, Northeastern University, Boston, MA 02115, USA; 2 Department of Chemistry, William Paterson University, Wayne, NJ 07470, USA; satamn@wpunj.edu

**Keywords:** biomolecules, biorthogonal, click chemistry, drug discovery, inhibitors, reactive fragments, target-guided synthesis

## Abstract

The rapid expansion of click chemistry reflects its transformative influence on contemporary drug discovery. This review highlights major advances in the application of click reactions within the kinetic target-guided synthesis (KTGS) paradigm for identifying potent inhibitors across a broad range of biological targets. KTGS constitutes a methodological shift that leverages the inherent dynamics of biomolecular systems, enabling biological targets to direct the in situ assembly of high-affinity bidentate ligands from a diverse repertoire of reactive building blocks. The review systematically classifies the principal bond-forming reactions that underpin effective inhibitor generation via KTGS. Collectively, it provides a comprehensive and scholarly analysis of how click-chemistry-enabled KTGS is redefining drug discovery and expediting the development of next-generation therapeutics.

## 1. Introduction

Recently, target-guided synthesis (TGS) [1,2,3,4,5,6,7,8,9,10] has been widely used to discover novel and selective small molecule binders of biological targets of interest from a pool of fragments bearing complementary reactive functional groups. TGS strategies are sometimes also referred to as target-selective synergism, receptor-accelerated synthesis, or target-driven chemistry [4]. The concept of TGS was initially introduced by Rideout et al. [11,12], who reported significant cytotoxic effects in cells attributed to the in situ-formed hydrazone, rather than the individual reactive components, namely decanal and *N*-amino-guanidines. Target-assembled products are likely to interact more strongly with the biological target than individual reactive building blocks, due to the bivalency of the in situ-formed compound. TGS represents a robust methodology for identifying inhibitors of biological targets by integrating synthesis and screening into a single, cohesive step. This approach has great potential to expedite the identification of drug candidates, as only the reactive building blocks, not all their combinations, need to be synthesized. The discovery of new compounds that modulate biological function is the primary focus of drug discovery programs. Over the past few years, fragment-based lead discovery has gained considerable popularity, as it enables more efficient sampling of chemical space, resulting in higher hit rates than screening non-fragment chemical libraries. TGS, an unconventional fragment-based lead discovery methodology, has a clear advantage over conventional approaches. It relies on the biological target to assemble its own bivalent modulators, which sets it apart from other methods. In theory, bivalent ligands bind to biological targets non-covalently with stronger affinity than the individual monovalent fragments due to synergism. TGS is a powerful fragment-based strategy that provides ligands with reduced molecular weight and higher ligand efficiency, thereby improving the cell permeability, bioavailability, and metabolic stability of lead candidates.

**Figure 1:**
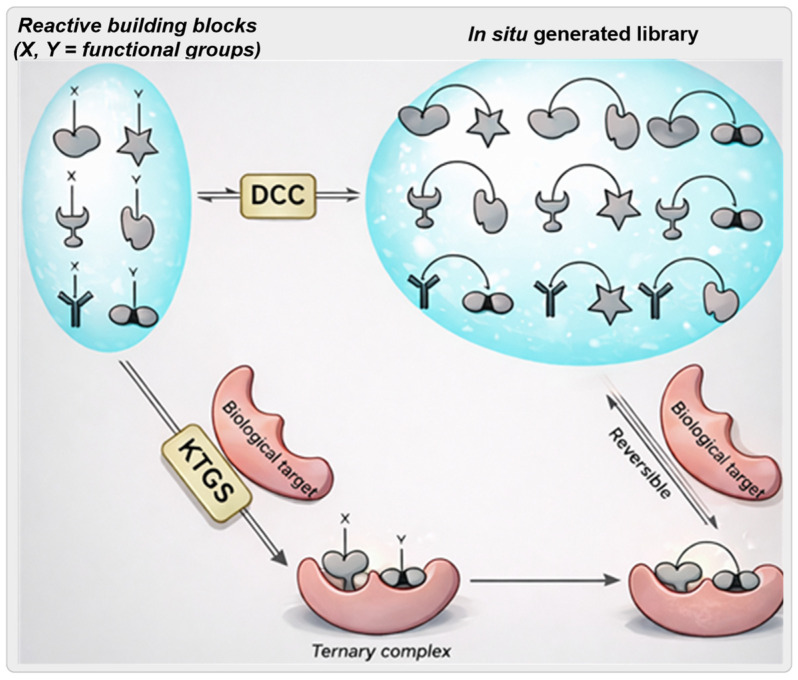
Schematic depiction of DCC and KTGS.

TGS can be categorized into the following two primary classes: (1) dynamic combinatorial chemistry (DCC) and (2) kinetic target-guided synthesis (KTGS). In DCC, the assembly process is reversible, allowing complementary reactive fragments to combine in various ways. Upon introducing a biological target, the equilibrium shifts towards the assembled product, demonstrating the highest affinity (Figure 1). Conversely, KTGS features an irreversible assembly process in which the biological target facilitates the formation of a ternary complex between the complementary reactive fragments and the target, enabling selectivity for certain products over others (Figure 1). DCC is a thermodynamically controlled reaction, initially introduced by Lehn and colleagues [13], while KTGS is a kinetically controlled reaction pioneered by Sharpless et al. [14]. Both DCC and KTGS offer significant potential in drug discovery; however, they remain largely underexplored.

KTGS, a fascinating research domain, explores the macromolecular complexity and dynamics of biological targets. One of its most compelling features is its efficiency, as it eliminates the need for prior synthesis and purification of library compounds or for biochemical evaluation of each final library member. This efficiency surpasses that of conventional combinatorial chemistry or fragment-based drug design, making KTGS a promising area of research. In the context of TGS, the reactions aimed at combining complementary reactive fragments must be biocompatible. Click reactions, which have gained significant attention in recent years, are characterized by their high selectivity and efficiency. These features enable them to operate within intricate biological environments while exhibiting minimal or no interactions with biological molecules, thereby enhancing their applicability in TGS and instilling confidence in their use. The criteria for a reaction suitable for KTGS differ considerably from those of DCC or traditional organic reactions. Ideally, complementary reactive fragments should engage in slow reactions that produce a stable covalent bond with minimal or no side products. Furthermore, these reactive fragments should retain stability in aqueous conditions and be compatible with biological targets. Observing a significant difference in reaction rates between the biomolecule-templated reaction and the blank reactions is also essential. KTGS and related protein-templated ligation strategies have been reviewed extensively in the literature [1,2,3,4,5,6,7,8,9,10], highlighting their potential for ligand discovery, fragment linking, and chemical biology applications.

KTGS offers two key advantages that distinguish it from conventional drug discovery approaches [10]. First, it does not require prior knowledge of the target’s three-dimensional (3D) structure, thereby enabling the exploration of previously unrecognized or transient binding conformations. Second, KTGS integrates chemical synthesis and biological screening into a single, streamlined process, allowing bioactive compounds to be identified before their isolation and traditional synthesis.

Despite its transformative potential, KTGS is not without limitations. One notable challenge is the risk of false negatives, which may arise if the templated products interfere unfavorably with the target or if prolonged reaction times induce structural or functional changes in the target that compromise its catalytic activity [10].

The success of KTGS critically depends on the careful selection of suitable chemical reactions. These reactions must be bioorthogonal—fully compatible with biological systems—and exhibit a substantial rate enhancement in the presence of the target compared with control conditions. To date, only a limited set of click reactions has been explored in KTGS, with the 1,3-dipolar cycloaddition being the most extensively employed. The popularity of 1,3-dipolar cycloaddition stems from its operational simplicity, well-established synthetic accessibility, and the stability of its complementary reactive fragments.

## 2. Click Reactions in Kinetic Target-Guided Synthesis (KTGS)

The review delves into the role of click reactions within the KTGS framework for identifying non-covalently bound inhibitors in situ. Table 1 illustrates the array of click reactions documented in the literature for effectively identifying non-covalently associated inhibitors and classified into four main types depending on the type of bond formation, viz, carbon-nitrogen (C-N) bond forming reactions, carbon-sulfur (C-S) bond forming reactions, carbon-carbon (C-C) bond forming reactions, and carbon-carbon/carbon-nitrogen (C-C/C-N) bond forming reactions. These reactions capitalize on the specificity and efficiency of click chemistry, rendering it an invaluable tool in drug discovery. In addition to the reactions detailed in Table 1, two notable reactions—reductive amination, as reported by Hirsh et al. [15], and the Groebke–Blackburn–Bienaymé (GBB) reaction reported by Van der Veken et al. [16]—have emerged as relevant click reactions in this context. However, these reactions are not included in the scope of this review because they do not benefit from a templated effect. A templated effect, a key factor in enhancing selectivity and binding affinity in KTGS applications, is absent in these reactions.

Advancements in click reactions in KTGS are not only paving new avenues but also sparking excitement and potential for the discovery of effective inhibitors tailored to specific biological functions. Table 2 outlines the biological targets used in KTGS to identify non-covalently bound inhibitors via click reactions. A brief overview of each biological target is provided to enhance understanding of their significance in drug discovery.

### 2.1. Carbon-Nitrogen (C-N) Bond Forming Reactions

The diverse reactions that form carbon–nitrogen (C-N) bonds are widely used as click reactions in the context of KTGS. The extensively used C-N bond-forming click reaction in KTGS is the alkyne-azide Huisgen cycloaddition. Additionally, various C-N bond-forming click reactions, including sulfo- or seleno-click amidation, conventional amidation, oxime ligation, and *N*-alkylation, have been utilized in both binary and multi-fragment KTGS to identify inhibitors of various biological targets.

#### 2.1.1. Alkyne-Azide Huisgen Cycloaddition (1,3-Dipolar Cycloaddition)

**Scheme 1:**
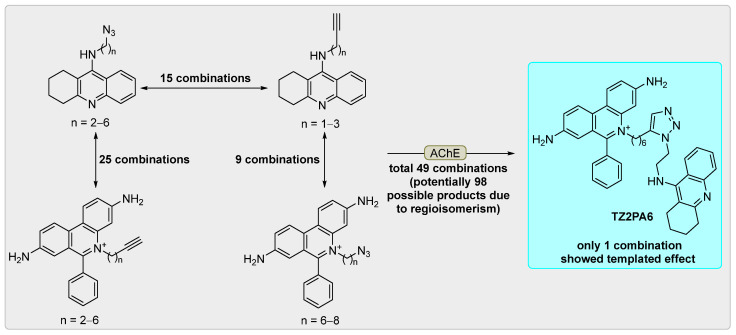
AChE-templated binary fragment KTGS.

Sharpless and colleagues first introduced alkyne-azide Huisgen cycloaddition in KTGS to identify potent inhibitors of acetylcholinesterase (AChE) from various building blocks (Scheme 1) [57]. Building upon the structural motifs of previously reported site-specific AChE inhibitors such as tacrine [58,59] and phenanthridinium [58,60], scaffolds containing alkyl azides and alkyl acetylenes of differing chain lengths were synthesized. Binary mixtures of all possible combinations were incubated in the presence and absence of AChE, and the resulting reaction mixtures were analyzed using DIOS-MS. Among the 49 possible combinations, only one—a triazole designated as **TZ2PA6**—was detected in the AChE-templated reaction, while no triazole was found in the AChE-free (blank) reactions. The formation of **TZ2PA6** was further verified through LC/MS analysis. A comparison of the HPLC traces of the in situ generated triazole with chemically synthesized *syn*- and *anti*-triazole (**TZ2PA6**) confirmed the selective formation of the *syn*-isomer in the AChE-templated reaction. The binding affinity of the *syn*-isomer was assessed using two techniques (stopped-flow and Ellman assay), revealing a strong affinity with *K_d_* values ranging from 77 to 410 fM. In contrast, the *K_d_* values for the corresponding complementary reactive fragments (**TZ2** and **PA6**) were significantly lower, ranging from 10 to 100 nM.

**Scheme 2:**
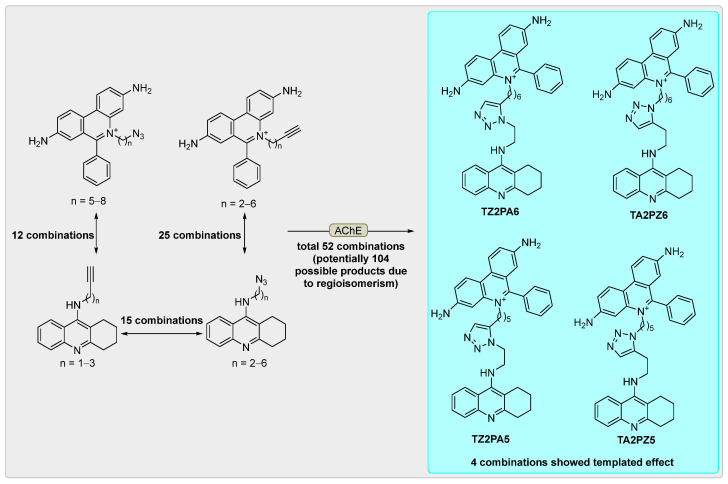
AChE-templated multi-fragment KTGS.

This approach was subsequently expanded to include 52 combinations to identify new AChE inhibitors (as illustrated in Scheme 2) [61]. The reaction mixtures were analyzed using a more sensitive LC/MS-SIM method for in situ formed products. The analysis revealed three new candidates—**TA2PZ6**, **TZ2PA5**, and **TA2PZ5**—in addition to the previously identified compound **TZ2PA6**. Moreover, the multi-fragment strategy was broadened to encompass the entire tacrine/phenylphenanthridinium library. The combinations were organized into five batches to streamline the identification of products with identical molecular weights. The findings indicated that, although multi-component screening was effective, it was associated with a slight reduction in sensitivity. In situ-generated triazoles were compared to chemically synthesized *syn*/*anti* triazoles using HPLC, with results showing the exclusive formation of *syn*-triazoles in the templated reaction. All identified hits were confirmed as AChE inhibitors, with dissociation constants in the femtomolar and picomolar ranges.

**Scheme 3:**
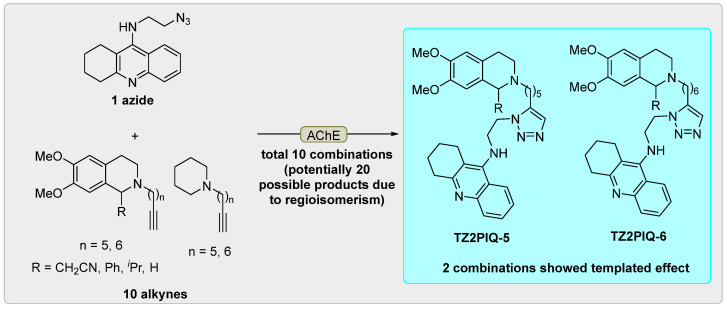
Discovery of AChE inhibitors via multi-fragment KTGS approach.

Subsequently, multi-fragment in situ click chemistry screening was employed, utilizing a tacrine building block as an anchor molecule. A library of acetylene reagents was designed to mimic the phenylphenanthridinium building block. AChE inhibitors were identified by incubating selected enzyme/tacrine azide combinations with a range of alkynes (Scheme 3) [62]. The reaction mixtures were analyzed using LC/MS-SIM, revealing the formation of only two products, **TZ2PIQ-5** and **TZ2PIQ-6**, in the presence of the target; no product formation was observed in control experiments. *Syn*-triazoles were selectively generated in the AChE-templated reaction, as determined by comparing the LC/MS-SIM traces of the in situ generated products with those of the chemically synthesized corresponding *syn*- and *anti*-triazoles. The hit compounds, **TZ2PIQ-5** and **TZ2PIQ-6**, demonstrated potent inhibition of AChE, with *K_d_* values of less than 40 fM.

**Scheme 4:**
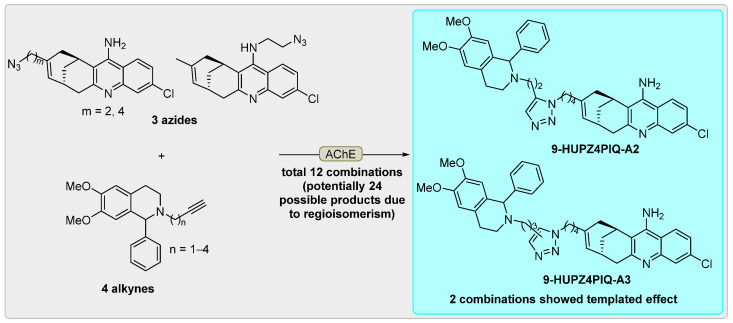
Identification of dual binding site AChE inhibitors via multi-fragment KTGS approach.

AChE-templated in situ synthesis of sub-nanomolar dual binding site inhibitors has been reported using a multi-component KTGS strategy (Scheme 4) [63]. In this study, AChE inhibitors containing Huprine with alkyl azides (previously reported Huprine-based AChE inhibitors) [64,65] and phenyl tetrahydroisoquinolines with alkyl acetylenes of varying lengths (with the phenyl tetrahydroisoquinoline motif serving as a helpful peripheral binder in TGS) [62] were incubated with and without AChE. The reaction mixtures were analyzed using LC/MS-SIM. Only the products **9-HUPZ4PIQ-A2** and **9-HUPZ4PIQ-A3** were observed in the templated reaction, while no product formation occurred in the control reaction. A comparison of the LC/MS traces of the in situ-synthesized products with those of chemically synthesized corresponding *syn*/*anti*-triazoles indicated the formation of *anti*-**9-HUPZ4PIQ-A2** and a regioisomeric mixture of *syn*- and *anti*-**9-HUPZ4PIQ-A3** (~1:2) in the templated reaction. The identified KTGS hits were potent inhibitors of AChE, with an IC_50_ of 0.6 ± 0.1 nM.

**Scheme 5:**
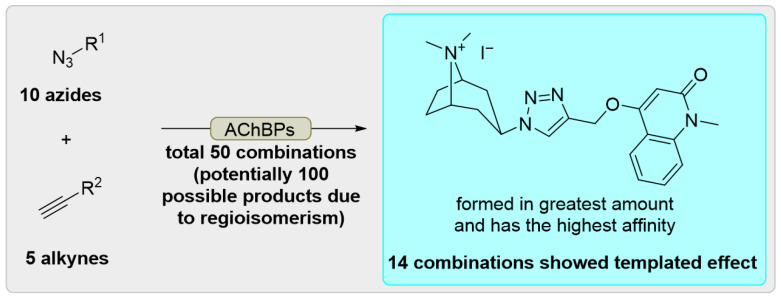
Multi-fragment KTGS to identify potent AChBPs inhibitors.

The acetylcholine binding proteins (AChBPs) templated in situ click chemistry approach was demonstrated to generate novel and potent ligands that bind selectively to AChBPs [66]. The level of triazole formation on the AChBP template corresponds with the affinity of the triazoles for the nicotinic ligand binding site and, therefore, can be used to identify the nAChR ligand. AChBPs templated in situ click chemistry approach has been demonstrated to generate novel and potent ligands that selectively bind to AChBPs. The extent of triazole formation on the AChBP template indicates these triazoles’ affinity for the nicotinic ligand binding site, making it a valuable tool for identifying ligands for nAChRs. AChBPs share homology with the extracellular domain of pentameric ligand-gated ion channels. Building on the hypothesis that 1,2,3-triazole can mimic the ester moiety of acetylcholine through its hydrogen bond acceptor characteristics (an interaction that has been previously reported) [67,68,69], a library of azides and alkynes was incubated simultaneously in the presence of AChBPs. The resulting reaction mixtures were analyzed using LC/MS-SIM. Reactions were also performed using Bovine Serum Albumin (BSA) as a control. The comparison of triazole formation in the presence of AChBPs to that of the control experiments revealed an accelerated triazole formation when AChBPs were present. The triazole (as illustrated in Scheme 5) was identified in the highest quantity and exhibited a strong affinity, with a *K_d_* of 0.96 ± 0.22 nM, as determined by scintillation proximity assay (SPA).

**Scheme 6:**
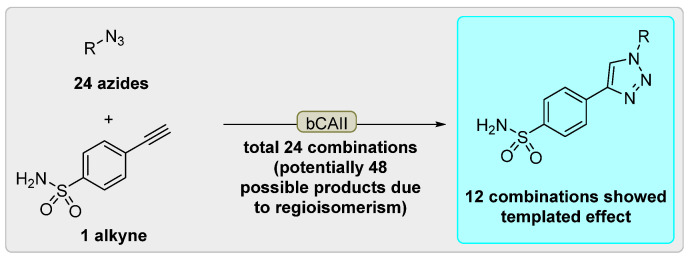
Discovery of CAII inhibitors by incubating binary mixtures.

In situ carbonic anhydrase II (CAII) inhibitor generation was achieved by reacting alkyne with various azides (Scheme 6) [70]. For this study, acetylenic benzenesulfonamide was chosen as a reactive scaffold, inspired by previously reported CA inhibitors [71,72,73,74,75], to capture complementary reactive fragments from a set of 24 azides, thereby forming divalent CA inhibitors in situ. The binary mixtures were incubated in the presence and absence of CAII, followed by analysis using LC/MS-SIM. The results indicated that out of the 24 combinations, 12 resulted in the formation of corresponding triazoles only in the presence of the enzyme. In contrast, no product formation occurred in enzyme-free conditions. Comparing the LC/MS traces of the in situ formed triazoles with those of chemically synthesized *syn*/*anti*-triazoles demonstrated that the enzyme-templated reaction was highly selective, yielding only *anti*-triazoles. The identified hits were chemically synthesized, and the dissociation constant (*K_d_*) was determined through a fluorescence-based assay. Notably, all products generated in situ exhibited higher binding affinities for bCAII (*K_d_* = 0.2–7.1 nM) compared to the individual reactive fragments (alkyne—*K_d_* = 37 ± 6 nM; azides—*K_d_* = high µM affinity).

**Scheme 7:**
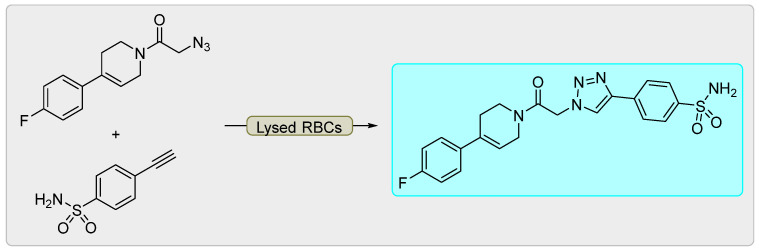
Cell-based KTGS.

Cell-based KTGS has been demonstrated using LC/MS-MS techniques. Previous studies reported [76] the in situ generation of triazole from azide [2-azido-1-(4-(4-fluorophenyl)-3,6-dihydropyridin-1(2H)-yl)ethan-1-one] and alkyne (acetylenic benzenesulfonamide), targeting bCAII as the biological component. Cell-based KTGS was conducted by reacting azide and alkyne in bovine blood (Scheme 7) [77], given that bCAII is a prevalent enzyme in red blood cells. Utilizing freeze-lysed packed red blood cells, the resulting triazole production was nearly comparable to that obtained with purified carbonic anhydrase II, and it significantly exceeded the amounts generated in control experiments without RBCs.

**Scheme 8:**
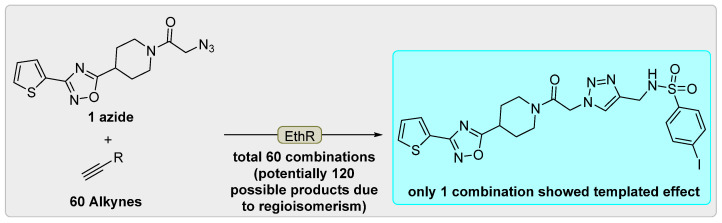
Multi-fragment KTGS using EthR as the biological target.

The in situ generation of triazoles, templated by EthR, was explored using one azide and six sets of acetylenic fragments, each containing 10 distinct fragments (Scheme 8) [78]. The azide was designed based on a previously reported EthR inhibitor, and 60 different alkynes were selected for their potential to interact with the hydrophobic portion of the ligand binding pocket. Reaction mixtures, comprising 1 azide and 10 alkynes, were incubated in the presence and absence of EthR and analyzed via LC/MS-SIM. Notably, only one combination exhibited the templated effect. The kinetic product of the reaction was identified as the 1,4-regioisomer, as confirmed by comparing the LC/MS traces of the templated product with those of the chemically synthesized 1,4- and 1,5-regioisomers. The hit ligand demonstrated a 10-fold increase in activity (IC_50_ = 580 nM) compared to the corresponding azide (IC_50_ = 7.4 µM), as assessed through an SPR assay.

**Scheme 9:**
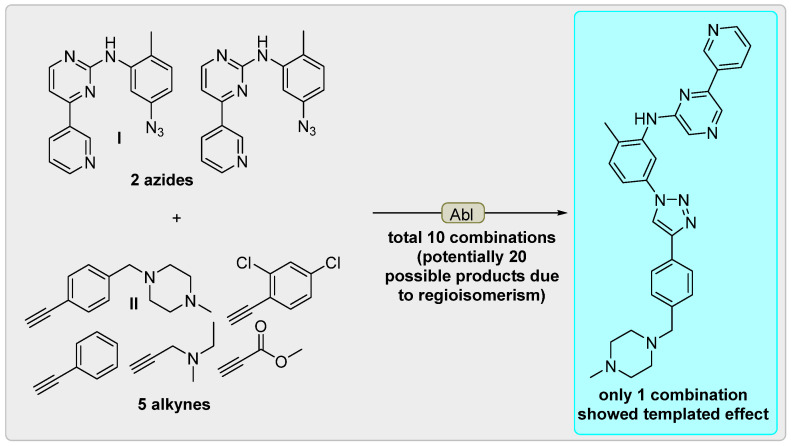
Abl-templated KTGS.

The in situ generation of triazoles using an Abl-templated approach from a combination of two azides and five alkynes aimed at identifying the most effective binding ligand was reported (Scheme 9) [79]. The azides and alkynes were incubated with and without Abl, followed by an HPLC and ESI-HRMS analysis. Azide **I** and alkyne **II** were previously identified [80] as scaffolds for synthesizing Bcr-Abl inhibitors. The HPLC and MS analyses revealed the formation of a single product out of the ten possible products in the templated reaction. In contrast, no product formation was observed in the control experiment.

**Scheme 10:**
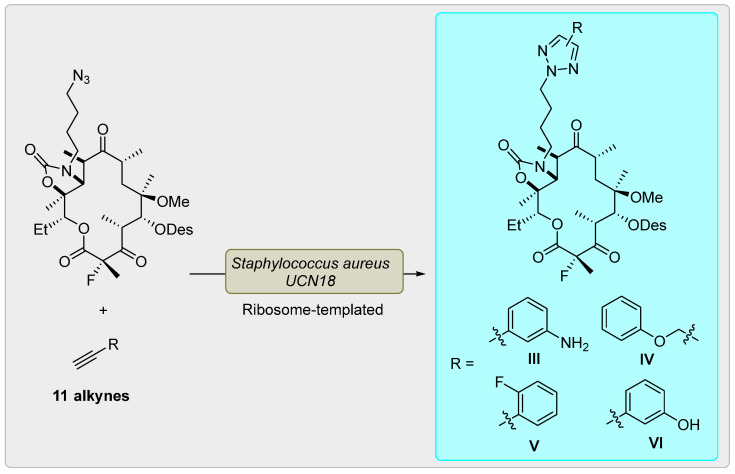
Ribosome-templated in-cellulo click chemistry.

Ribosome-templated in-cellular click chemistry was demonstrated, wherein bacterial cells served as reaction vessels (Scheme 10) [81]. Potent macrolide antibiotics were reported previously [82] by reacting macrolide azide and alkyne via in situ click chemistry using a bacterial ribosome as the target. Multi-component in cellular click chemistry was employed by reacting macrolide azide with 11 alkynes. As a control experiment, the reactions were performed without a bacterial strain. The formation of triazoles was confirmed by LC/MS analysis and by comparison with the chemically synthesized triazoles. Of the 11 triazoles formed in-cellular click chemistry, triazole **III** (solithromycin) was formed in the highest amount with a MIC of 2 µg/mL, followed by triazoles **IV** (4 µg/mL), **V** (8 µg/mL), and **VI** (8 µg/mL).

**Scheme 11:**
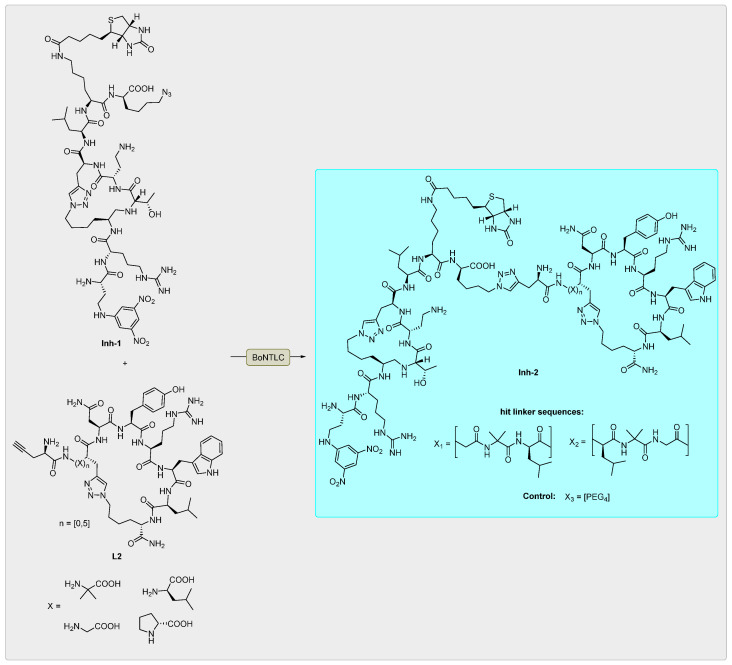
BoNTLC-mediated KTGS to form divalent **Inh-2**.

The strategic utilization of the tertiary structure of the protein target Botulinum neurotoxin serotype A (BoNT/A) was highlighted as a framework for assembling a divalent ligand through in situ click chemistry [83]. A macrocyclic peptide ligand (**Inh-1**, *K_d_* = 68 ± 29 nM) was developed to serve as a substrate mimic for BoNT. An in situ click screening approach targeting epitopes [84] was employed to identify a second macrocyclic peptide (**L2**, *K_d_* = 78 ± 13 nM). The BoNTLC in situ click screening template was used to discover linear peptides that connect the two macrocycles (Scheme 11). **Inh-1** was synthesized with a C-terminal alkyne and the linker library of oligopeptides (ranging from 0 to 5 units) on Tentagel resin. A divalent ligand (**Inh-2**) emerged as a promising hit candidate. This identified hit was chemically synthesized and demonstrated potent inhibition of BoNTL, with an IC_50_ of 165 ± 15 pM as measured in vitro using a FRET-based substrate cleavage assay. Furthermore, **Inh-2** was tested for its capacity to protect and rescue human neurons from BoNT intoxication, successfully inhibiting the holotoxin in live cells.

**Scheme 12:**
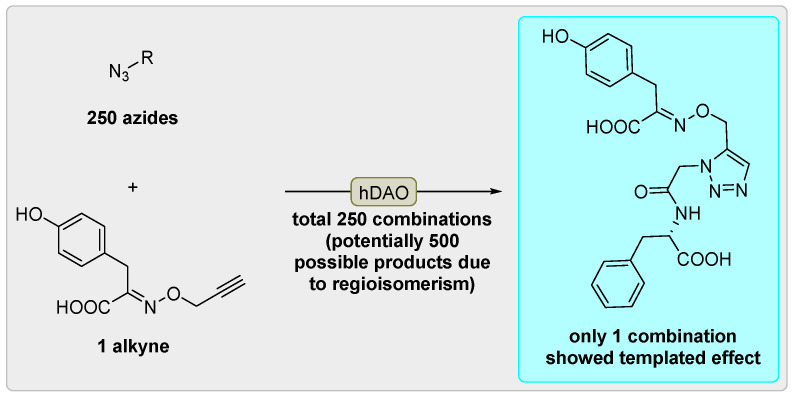
Identification of DAO inhibitor via KTGS.

The in situ generation of a D-amino acid oxidase (DAO) inhibitor was achieved by reacting a tyrosine-type alkyne with 250 diverse azides (Scheme 12) [85]. Utilizing the X-ray co-crystal structure of hDAO [86,87,88,89,90], an anchor molecule featuring an alkyne was designed and synthesized as a reactive scaffold to capture complementary azide fragments through click chemistry. In situ, click chemistry screening, which involved the reaction of the tyrosine-type alkyne with various azides in the presence and absence of hDAO, followed by LC/MS-SIR analysis, revealed that only one combination exhibited a templated effect. A comparison of the LC/MS traces of the in situ formed triazole with chemically synthesized syn/*anti*-triazoles indicated that the presence of hDAO favored the formation of a selectively *syn*-triazole. This *syn*-triazole demonstrated significantly higher inhibitory activity against hDAO, with a *K_i_* value of 0.5 mM measured through an oxygraphic assay compared to the individual reactive fragments.

**Scheme 13:**
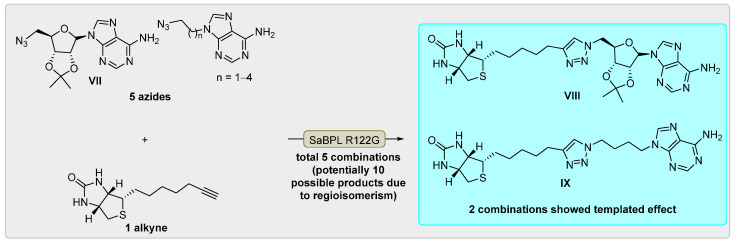
Multi-fragment SaBPL-templated reaction.

A multi-component in situ click approach for identifying inhibitors of biotin protein ligase (BPL) has been outlined (Scheme 13) [91]. Triazole **VIII** was identified as a potent and selective inhibitor of BPL derived from the pathogenic bacterium *S. aureus* [92]. The SaBPL enzyme was chosen as the ideal biological target for in situ click chemistry due to its active site, which features two well-defined pockets. An alkyne was reacted with a small library of azides, both in the presence of and absent from mutant SaBPL, to facilitate the in situ generation of triazoles. Azide **VII** served as a reference, while azides lacking a furanose moiety were explicitly designed to assess the significance of the furanose ring and the spacer length on biological potency. The reaction mixture was analyzed using LC/MS-SIM. The results demonstrated the formation of triazoles **VIII** and **IX** in the presence of SaBPL R-122G, while no product was detected in control experiments. Triazole **IX** emerged as the major product and exhibited the highest potency with a *K_i_* value of 0.66 ± 0.005 µM, indicating that the furanose moiety present in **VIII** (*K_i_* = 1.83 ± 0.033 µM) is not essential for activity. An appropriate spacer between the triazole and adenine group, as seen in **IX**, is crucial for maintaining activity.

**Scheme 14:**
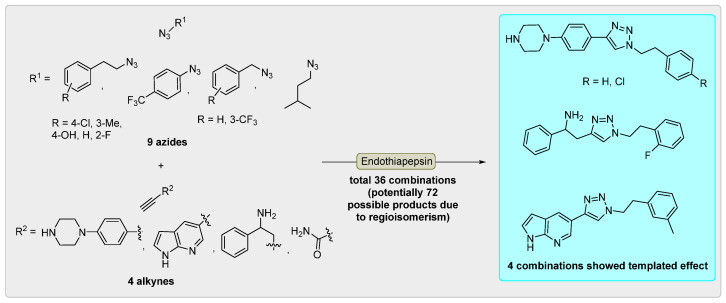
Aspartic protease endothiapepsin-templated click chemistry.

The strategic integration of fragment linking and optimization with protein-templated click chemistry was exemplified using the aspartic protease endothiapepsin as the biological target (Scheme 14) [93]. A range of azides and alkynes was designed by utilizing the X-ray crystal structure [94] of endothiapepsin in complex with various fragments. In situ click chemistry was performed in both the presence and absence of endothiapepsin, involving the reaction of alkynes with azides, followed by analysis via UPLC-TOF-SIM. This approach led to the identification of four 1,4-triazoles that were detected only in the presence of the target protein. All four identified triazoles were subsequently chemically synthesized, and their inhibitory activities were assessed using a fluorescence-based assay. Among the four triazoles, three inhibited endothiapepsin, with IC_50_ values ranging from 43 to 121 µM.

**Scheme 15:**
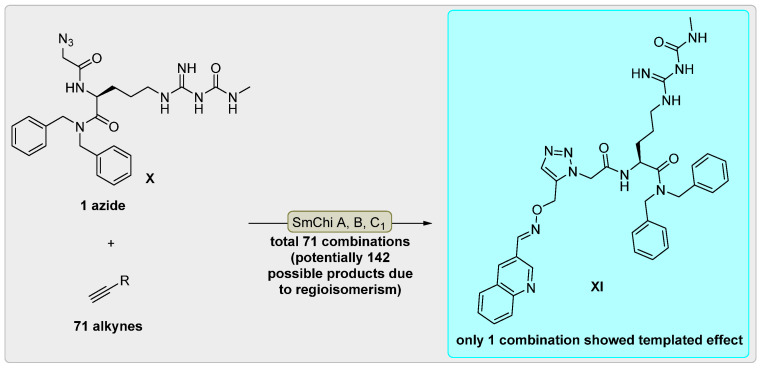
Multi-fragment SmChi-templated KTGS.

A highly potent chitinase inhibitor was discovered through in situ click chemistry by reacting azide **X** with various structurally diverse alkyne (Scheme 15) [95]. Azide **X** bearing *N*ω-methyl carbamoyl-L-arginine substrate was designed based on a macrocyclic peptide natural product, argifin (a potent inhibitor of Serratia marcescens chitinases [SmChi]) [96,97,98,99] and then used the TGS approach to identify a more potent inhibitor of SmChi by reacting with a library of alkynes. Azide **X** and 71 structurally diverse alkynes were incubated with and without a mixture of SmChi A, B, and C1 and analyzed by LC/MS-SIR (LC/MS-SIM). The results indicated that only one product, triazole **XI**, was detected in the presence of SmChi, and no product was detected in the blank experiment. By comparing the inhibitory activities of the in situ formed product with the chemically synthesized *syn*- and *anti*-triazole, it was found that only the *syn*-isomer was formed in the templated reaction. Triazole **XI** displayed high inhibitory activities against SmChi (IC_50_ = 0.045 µM for SmChiA and C1), similar to that of azide **X** but 10-fold higher than argifin while for SmChi B, IC_50_ = 0.022 µM.

**Scheme 16:**
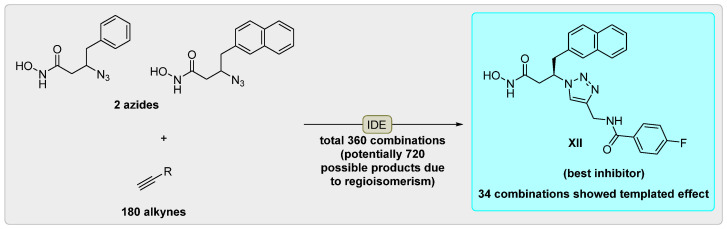
Discovery of IDE inhibitors via multi-fragment KTGS.

The discovery of inhibitors for the insulin-degrading enzyme (IDE) utilizing multicomponent KTGS reactions has been successfully demonstrated by Deprez-Poulain and colleagues [100]. In situ, click chemistry was employed to generate divalent inhibitors using two azides with hydroxamate warheads, which were specifically designed to bind to the catalytic zinc ion of IDE [41]. Additionally, a range of diverse alkynes was selected as potential binders for the C-terminal region of the catalytic cycle (Scheme 16). Among the various triazoles produced under KTGS conditions, triazole **XII** emerged as the most effective inhibitor. Furthermore, structure–activity relationship (SAR) analyses, inspired by the TGS experiments, were conducted to identify even more potent IDE inhibitors. These SAR studies confirmed that triazole **XII** remained the most active compound. X-ray crystallography was employed to determine the binding conformation of triazole **XII** to human IDE (hIDE) to elucidate its structure. Finally, the in vivo effects of **XII** on exogenous insulin were assessed through an insulin tolerance test (ITT) in mice, which demonstrated that triazole **XII** effectively increased the phosphorylation of insulin receptors in both liver and muscle tissues.

**Scheme 17:**
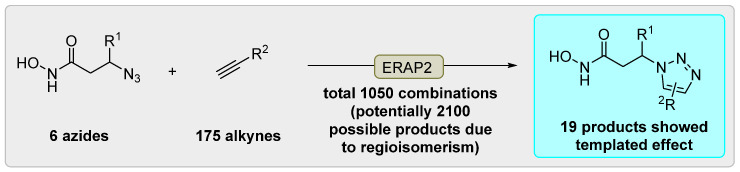
Multi-fragment KTGS to identify inhibitors of the ERAP2.

Further, Deprez-Poulain’s group has extended a multi-fragment KTGS approach by utilizing six azides and 175 alkynes to identify inhibitors of the enzyme ERAP2 (Scheme 17) [101]. This methodology features azides that incorporate hydroxamic acid as a zinc-binding group and were selected from their in-house library to evaluate ERAP2 inhibition. To enhance diversity, the extensive library of 175 alkynes is organized into 18 clusters, each consisting of nine or 10 distinct alkynes. A 1,3-dipolar cycloaddition was performed with the azides containing a hydroxamic acid. In total, 72 mixtures of alkyne and azide, at a ratio of nine or ten to one, were incubated with ERAP2, potentially yielding up to 2100 products, which include 1,4- and 1,5-disubstituted regioisomers of 1,2,3-triazoles, followed by analysis using LC-MS/MS. Among the 19 identified ERAP2 hits, nine demonstrated an IC_50_ value of less than 25 µM. The elucidation of structure–activity relationships for these KTGS hits facilitated the discovery of the first nanomolar inhibitor of ERAP2.

**Scheme 18:**
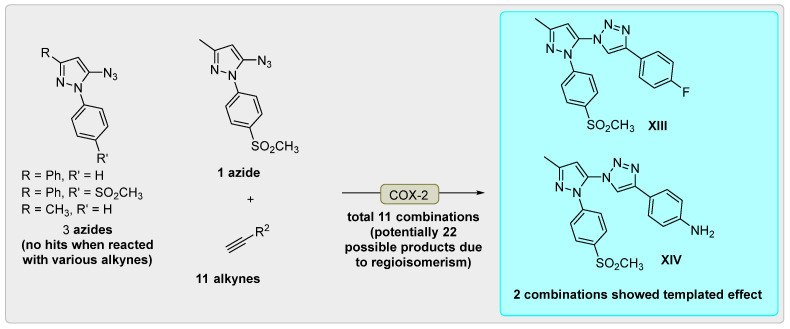
Discovery of the COX-2 inhibitor.

Two highly potent and selective inhibitors of the cyclooxygenase-2 (COX-2) isozyme were identified using the KTGS approach from a library of azides and alkynes. A series of pyrazole-based azide building blocks were designed, synthesized, and subsequently reacted with various aryl alkynes, both in the presence and absence of COX-2, to assess the isozyme’s ability to assemble its own divalent inhibitors (Scheme 18) [102]. The reaction mixtures were analyzed using LC/MS-SIM, revealing the formation of two triazoles, designated **XIII** and **XIV**, in the presence of COX-2. In contrast, no product formation occurred in the control experiments. A comparison of the LC/MS traces of the in situ formed products with chemically synthesized corresponding 1,4/1,5-triazoles confirmed the exclusive formation of the 1,4-triazole regioisomer in the templated reaction. **XIII** and **XIV**’s in vitro inhibitory activities were evaluated, demonstrating potent effects with IC_50_ values of 0.09 µM and 0.05 µM, respectively. Furthermore, both compounds’ in vivo anti-inflammatory activities were assessed, and they proved to be highly effective anti-inflammatory agents.

**Scheme 19:**
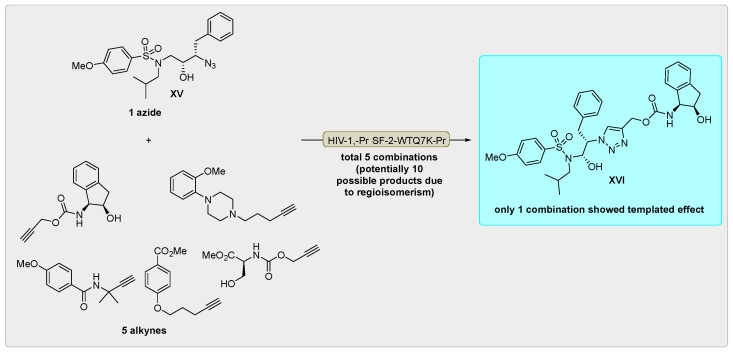
Multi-fragment KTGS to identify inhibitors of the wild-type HIV-1-Pr.

An in situ click chemistry strategy was employed to develop inhibitors of HIV-1 protease (HIV-1-Pr), as illustrated in Scheme 19 [103]. The incubation of a mixture containing azide **XV** and five different alkynes, both in the presence and absence of HIV-1-Pr SF-2_WTQ7K-Pr, was analyzed using LC/MS-SIM. The results indicated the formation of only one triazole in the presence of the enzyme, while no product was detected in its absence. By comparing the LC/MS traces of the in situ generated product with those of chemically synthesized *syn*- and *anti*-triazoles, it was determined that the templated reaction produced *anti*-triazole **XVI**. This compound was previously characterized as an inhibitor of wild-type HIV-1-Pr, exhibiting an IC_50_ of 6 nM and a *K_i_* of 1.7 nM.

#### 2.1.2. Sulfo- or Seleno-Click Amidation

**Scheme 20:**
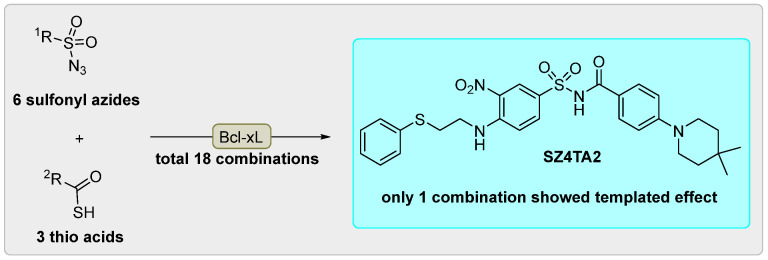
Bcl-xL-mediated sulfo-click amidation.

The initial investigation into the application of KTGS for targeting protein–protein interactions (PPIs) through a sulfo-click amidation reaction involving thio acids and sulfonyl azides was conducted by the research team led by Manetsch. This effort involved the synthesis of a library of complementary fragments, comprising thio acids and sulfonyl azides, aimed at identifying a potent inhibitor of the Bcl-xL protein utilizing the KTGS methodology (Scheme 20) [104]. The thio acids and sulfonyl azides were incubated in a binary mixture of 18 distinct combinations, each containing one thio acid and one sulfonyl azide, both in the presence and absence of the Bcl-xL protein. The resulting products were subsequently analyzed using LC/MS-SIM to assess the formation of acylsulfonamides. Among the 18 combinations tested, only one demonstrated a templated effect. The KTGS hit compound, **SZ4TA2**, was chemically synthesized and subjected to a fluorescence polarization competition assay to evaluate its efficacy in inhibiting the interaction between Bcl-xL and Bak. The outcomes indicated that the KTGS hit compound effectively inhibited the Bcl-xL and Bak interaction, yielding an IC_50_ value of 78.8 nM.

**Scheme 21:**
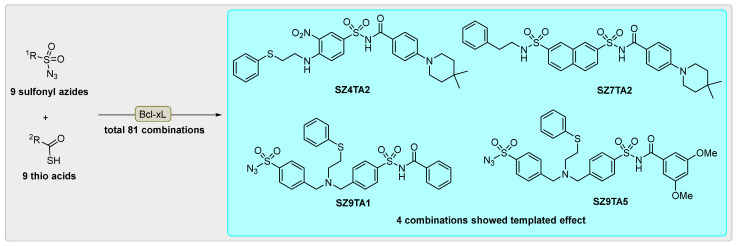
Bcl-xL templated sulfo-click amidation.

The Bcl-xL templated sulfo-click amidation was expanded to investigate a wider array of reactive fragments from the compound library to identify novel inhibitors of protein–protein interactions (PPIs). In this study, a binary mixture composed of nine thio acids and nine sulfonyl azides was incubated and subsequently analyzed using LC/MS-SIM to facilitate the formation of acylsulfonamides (Scheme 21) [105]. Only four of 81 potential combinations exhibited the templated effect associated with Bcl-xL. This work identified three new compounds (**SZ7TA2**, **SZ9TA1**, and **SZ9TA5**), which were synthesized alongside a previously identified hit compound (**SZ4TA2**). The chemical properties of the three compounds were evaluated for their ability to modulate PPI through a fluorescence-based competitive binding assay. The results demonstrated that all identified KTGS hits displayed significant modulatory activities on PPI.

**Scheme 22:**
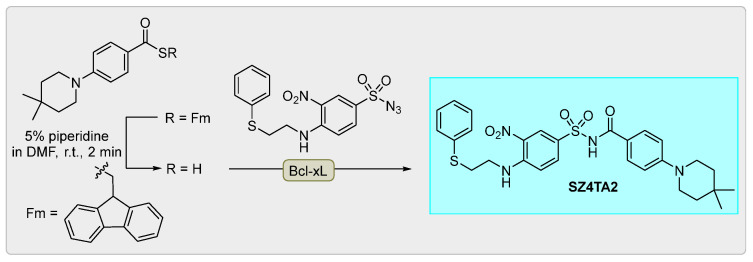
Sulfo-click amidation via in situ generated thio acid and its application in KTGS.

While the sulfo-click amidation method has proven its reliability in various applications, its broader adoption has been limited due to the challenges in preparing and handling thioacids. However, Manetsch’s research group has successfully addressed this issue by implementing a practical one-pot deprotection/sulfo-click amidation strategy within the framework of KTGS. This innovative approach involves the in situ generation of thio acids from the corresponding 9-fluorenylmethyl (Fm) thioesters and sulfonyl azide, followed by sulfo-click amidation, with Bcl-xL serving as the biological target (Scheme 22) [106]. The Fm-thioesters were treated with 5% piperidine in DMF to generate the thio acid in situ, which was then reacted with sulfonyl azide in the presence of Bcl-xL, leading to the formation of the desired sulfonyl amide. The reaction was analyzed using LC/MS-SIM, confirming the successful formation of the target product. This study utilized previously reported [105]. KTGS hit fragments to illustrate the practicality and effectiveness of this one-pot deprotection/amidation protocol within KTGS.

**Scheme 23:**
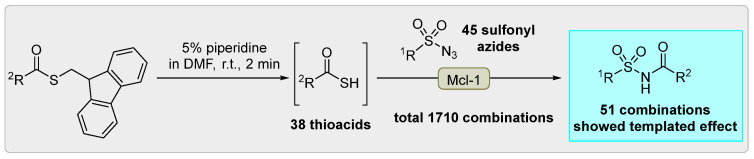
Mcl-1-templated multi-fragment KTGS.

The sulfo-click KTGS methodology has been significantly enhanced by implementing LC-MS/MS detection, allowing for the most comprehensive multi-fragment KTGS screening reported to date [107]. The optimized multi-fragment KTGS strategy facilitated the examination of 1710 potential fragment combinations across 18 wells of a 96-well plate, with 190 fragment combinations assessed in a single well. Of these, nine wells contained the protein target, while the other nine were devoid of it. An 83-member fragment library was employed in both the presence and absence of Mcl-1 and subsequently analyzed using LC-MS/MS. Among the 51 identified hits (Scheme 23), the investigation successfully pinpointed 24 Mcl-1 inhibitors exhibiting single-digit micromolar IC_50_ values, with these findings validated through fluorescence polarization (FP) studies. These results highlight the efficacy of the multi-fragment sulfo-click KTGS approach in developing hit compounds targeting Mcl-1. This discovery represents a significant breakthrough in KTGS screening, documenting the highest number of unique combinations per well ever recorded and opening new avenues for research in fragment-based lead discovery via KTGS.

**Scheme 24:**
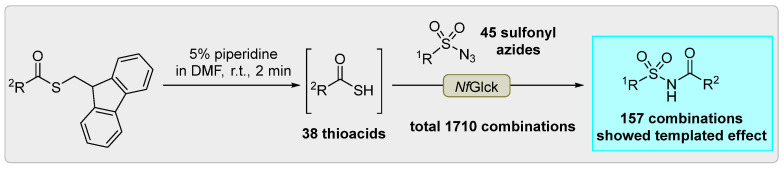
*Nf*Glck-templated multi-fragment KTGS.

The previously established multi-fragment KTGS screening method utilizing sulfo-click amidation was next employed to identify inhibitors of the glucokinase (Glck) enzyme derived from pathogenic free-living amoebae (pFLA). An in-house library of 83 fragments, including 38 in situ formed thioacids and 45 sulfonyl azides, identified 157 hit compounds through the multi-fragment KTGS approach (Scheme 24) [108]. Among these 157 hit compounds, twelve specific inhibitors targeting the pFLA Glck enzymes were discovered. The fragments were not chosen based on predetermined binding potentials, demonstrating that KTGS, alongside randomly designed fragments, can effectively screen chemical space and discover new compounds with medicinal chemistry potential.

**Scheme 25:**
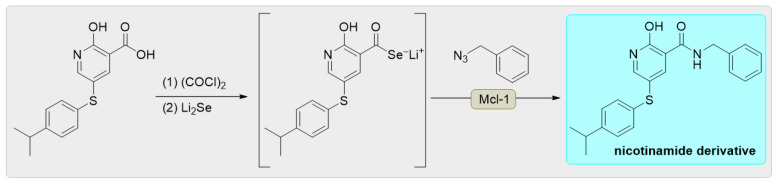
Binary fragment KTGS via Mcl-1-templated seleno-click amidation.

Subsequently, Manetsch’s group showcased the application of seleno-click amidation in the context of KTGS, using Mcl-1 as the biological target (Scheme 25) [109]. This proof-of-concept study successfully synthesized nicotinamide derivative **1**, a previously documented Mcl-1 inhibitor, from the corresponding reactive fragments. The templation effect was thoroughly examined and confirmed through LC-MS/MS analysis. The findings indicate that the significantly enhanced nucleophilicity of in situ-generated selenocarboxylates, compared to their sulfur counterparts, can facilitate amidation reactions with less reactive electron-rich azides. This makes it a compelling bioorthogonal reaction for applications in the KTGS field. Seleno-click amidation in KTGS was successfully performed not only at 37 °C but also at 4 °C (a temperature at which most biological targets remain stable for prolonged periods). This advancement significantly underscores the potential applications of seleno-click amidations within the KTGS framework.

#### 2.1.3. Conventional Amidation

**Scheme 26:**
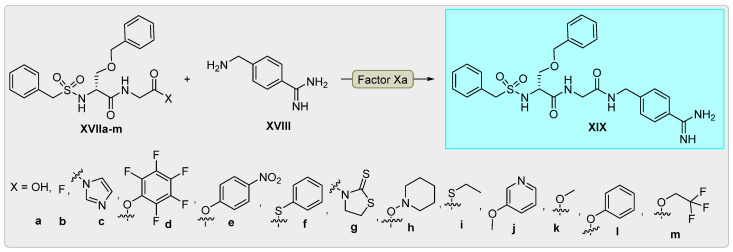
Factor Xa-assisted background-free amidation.

Background-free amidation reactions between activated carboxylic acids and nucleophilic amines were utilized for the protein-templated generation of an inhibitor of factor Xa (Scheme 26) [110]. Fragment **XVIIa** (where X = OH, specifically the dipeptide *O*-benzyl-*N*-benzyl-sulfonyl-D-serinyl-glycine) is a known inhibitor anticipated to bind effectively within the S2–S4 pockets of the enzyme [111]. By activating the carboxylic acid of fragment **XVII**, a library of compounds **XVIIa-m** was synthesized and subsequently reacted with the complementary reactive fragment **XVIII** (4-amino-methylbenzamidine, a typical S1-binding fragment of trypsin-like serine proteases). The binary mixture comprising fragment **XVIIa-m** and fragment **XVIII** was incubated in the presence and absence of protein factor Xa to examine the protein-templated formation of inhibitor **XIX**. The inhibition of protein factor Xa was quantified using the fluorogenic substrate 7-(*N*-Boc-leucinyl-glycinyl-arinyl)-7-amino-4-methylcoumarin via an enzyme activity assay. The active carboxylic acid derivatives **XVIIl** and **XVIIm** exhibited a noteworthy templating effect, as determined by an unpaired *t*-test. The validity of the template effect was confirmed through reverse-phase LC-MS analysis, which detected amidation product **XIX** within the binary incubation mixture containing the target protein. In contrast, no product formation was observed in the protein-free condition.

#### 2.1.4. Oxime Ligation

**Scheme 27:**
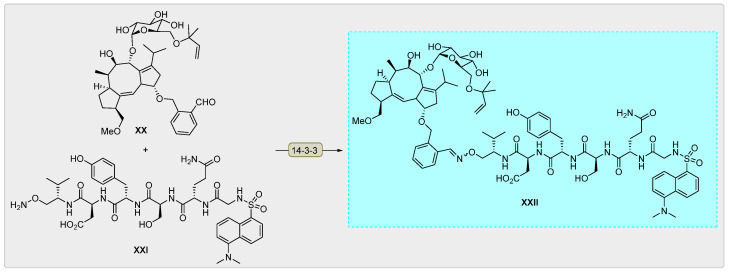
14-3-3-mediated oxime ligation.

Parvatkar et al. explored a rational strategy to generate inhibitors of 14-3-3-mediated protein–protein interactions through oxime ligation. Utilizing the co-crystal structure of the ternary complex formed by the diterpene natural product fusicoccin A, the PMA2 phosphopeptide QSYpTV, and the 14-3-3 protein, module compounds **XX** and **XXI** were designed. These compounds possess complementary reactive functional groups suitable for oxime ligation. A binary mixture of **XX** and **XXI** was incubated to generate the conjugate product in situ (Scheme 27) [112]. The in vitro oxime ligation of **XV** and **XXI** in a 14-3-3 free environment was notably slow, producing only a small amount of product **XXII**. In contrast, in the presence of 14-3-3, the reaction occurred swiftly—about 50 times faster than in the absence of 14-3-3. Over 90% conversion was achieved within 4 h with 14-3-3 present, while less than 10% conversion was noted in the control reaction, as determined by HPLC analysis. The chemically synthesized product **XXII** demonstrated a higher affinity for 14-3-3 (*K_d_* = 0.37 ± 0.14 µM) than the individual module compounds **XX** and **XXI**, as confirmed by an isothermal titration calorimetry experiment. Oxime ligation was subsequently conducted intracellularly using HEK293 cells that overexpressed FLAG-14-3-3. HPLC analysis indicated the formation of **XXII**, likely due to the templated effect of endogenous 14-3-3. Furthermore, a co-immunoprecipitation experiment revealed that **XXII** effectively inhibited the interaction between 14-3-3 and its partner protein, c-Raf.

#### 2.1.5. N-Alkylation

**Scheme 28:**
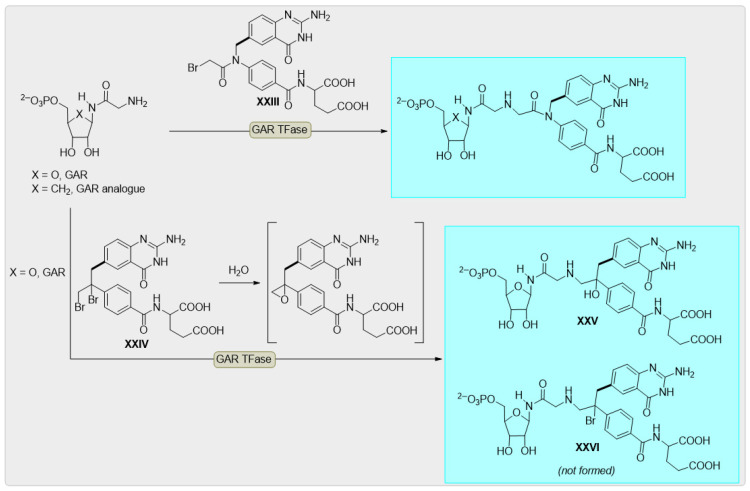
GAR TFase-templated *N*-alkylation.

The synthesis of the multi-substrate adduct inhibitor (MAI) of glycinamide ribonucleotide transformylase (GAR TFase) was accomplished by alkylating glycinamide ribonucleotide (GAR) with the bromo compound (Scheme 28) [113,114,115]. The incubation of binary mixtures comprising GAR, its analog, and *N*-10-(bromoacetyl)-dideazafolate in the presence of GAR TFase resulted in the formation of the corresponding *N*-alkylated products, as confirmed through HPLC isolation and spectroscopic analysis. The chemically synthesized *N*-alkylated products demonstrated substantial inhibitory activity, with *K_d_* values of 250 and 110 pM, respectively. However, the high reactivity and instability of the bromoacetyl group in bromo compound **XXIII** made it an unsuitable candidate for drug development [116]. To overcome this limitation, a less reactive analog of **XXIII**, dibromide **XXIV**, was designed, synthesized, and subsequently reacted with GAR in the presence of GAR TFase. X-ray analysis of the co-crystal structure of the resulting product with GAR TFase revealed the unexpected formation of compound **XXV** instead of the anticipated compound **XXVI**. This unforeseen product likely arose from the reaction of GAR with an in situ-formed epoxide from the dibromide via a bromonium ion, followed by the addition of water. The time-dependent inhibitory activity of dibromide and epoxide against GAR TFase was observed in the presence of the substrate β-GAR; however, this inhibitory effect was lost in the absence of β-GAR, underscoring the critical role of the substrate in the formation of MAI.

### 2.2. C-S Bond Forming Reactions

In the context of KTGS, a variety of effective click reactions that facilitate the formation of carbon–sulfur (C-S) bonds are employed to identify inhibitors of the biological target of interest. These reactions include S-alkylation, thiol-mediated opening of epoxide rings, thio-Michael addition, thiol-yne addition, and the photochemical reaction of diazirine with thiols. Each reaction plays a significant role in the systematic identification and development of potential inhibitors.

#### 2.2.1. S-Alkylation

**Scheme 29:**
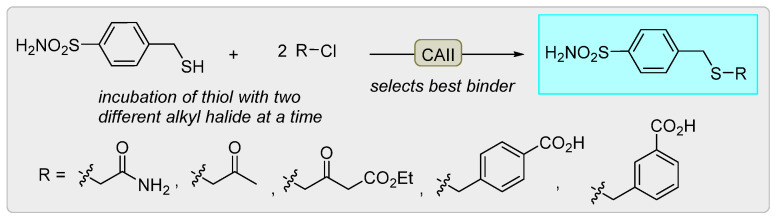
CAII templated *S*-alkylation.

The synthesis of an inhibitor for carbonic anhydrase II (CAII) was explored through a competition assay involving the reaction of α-mercaptotosylamide with various alkyl chlorides (Scheme 29) [117]. The findings revealed that products with more hydrophobic substituents at the para position exhibited enhanced inhibitory activity, with inhibition constants (*K_i_*) ranging from 770 to 59 nM, as determined by spectrophotometric assays. It is well-established that *para*-substituted aromatic sulfonamides [13] possess a notable binding affinity for CAII. By introducing thiol groups into these sulfonamides, *S*-alkylation was performed using different alkyl halides. The hydrophobic para substituents of the aromatic sulfonamides engage with the hydrophobic wall of the CAII protein, thereby improving their binding affinity. In this study, two alkyl halides were reacted simultaneously with α-mercaptotosylamide, producing two distinct compounds. The compound with greater hydrophobicity in its *para* substituents displayed a more significant templating effect, as analyzed by HPLC. Ultimately, CAII demonstrates a strong preference for the most effective inhibitors.

#### 2.2.2. Epoxide Ring Opening by Thiol

**Scheme 30:**
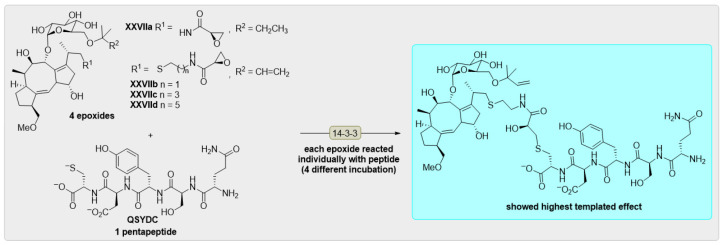
Binary fragment KTGS using 14-3-3 protein as the biological target.

The templated effect of 14-3-3 protein on the chemical ligation of fusicoccin containing an epoxide functional group and the pentapeptide QSYDC has been reported by Okhanda’s group (Scheme 30) [118]. Utilizing the co-crystal structure of the ternary complex formed by the natural product fusicoccin, the pentapeptide QSYpTV, and the 14-3-3 protein, compounds **XXVIIa-d** (fusicoccin with an epoxide group) and the pentapeptide QSYDC were designed and synthesized. Each epoxide (**XXVIIa-d**) was individually reacted with peptide QSYDC in both the presence and absence of 14-3-3 and the reaction progress was monitored via HPLC. Among the combinations tested, **XXVIIb** and QSYDC exhibited the highest templated effect, with the efficiency of product formation due to **XXVIIb** increasing to 199% in the presence of 14-3-3. In contrast, the efficiency of conjugate generation diminished with the longer (**XXVIIc-d**) and shorter (**XXVIIa**) spacers, highlighting the significance of spacer length in positioning the epoxide close to the thiol group of QSYDC. The epoxide opening chemical ligation presents a promising application for the in situ generation of PPI inhibitors.

#### 2.2.3. Thio-Michael Addition

**Scheme 31:**
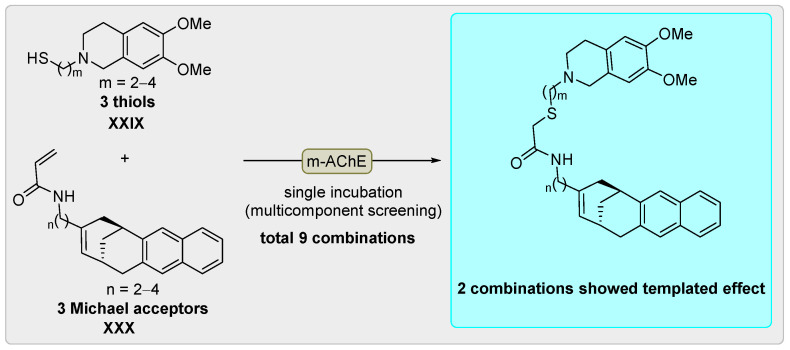
Multi-fragment KTGS using m-AChE as the biological target.

Thio-Michael addition to acrylamide derivatives has been demonstrated to create AChE inhibitors through the multi-fragment KTGS approach (Scheme 31) [119]. Previous research [63] has indicated that both Huprine (**XXX**) and 6,7-dimethoxy-1-phenyl-1,2,3,4-tetrahydroisoquinoline (**XXIX**) are effective in targeting the catalytic and peripheral sites of AChE. Molecular docking studies have shown that the thio-Michael addition is favorable when reactive fragments (**XXX** and **XXIX**) are equipped with acrylamide and thiol functional groups. A library of complementary reactive fragments was synthesized by varying the spacer length and tested in the presence and absence of AChE. In particular, no product formation was observed in the absence of AChE, while two products were generated in its presence, as identified by LC-MS out of nine potential products. The hits identified through the multi-component KTGS approach exhibited enhanced inhibitory activities compared to the individual reactive building blocks.

**Scheme 32:**
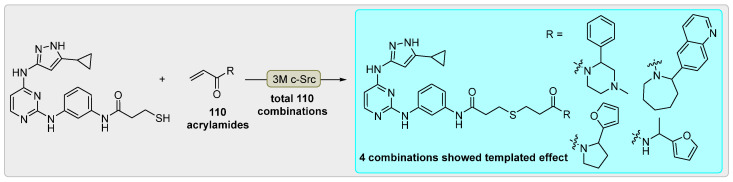
Binary fragment KTGS using 3M c-Src as the biological target.

Soellner’s research team utilized a c-Src (cellular Src kinase)-templated thio-Michael addition approach to discover bivalent inhibitors (Scheme 32) [120]. They introduced thiol groups to a previously established aminopyrazole inhibitor [121] and performed three mutant c-Src-templated screens to identify acrylamides capable of forming bivalent inhibitors. The resulting assembled bivalent inhibitors exhibited enhanced potency and selectivity compared to the initial fragments. Only four of the 110 potential combinations tested demonstrated a templated effect, with inhibition constants (*K_i_*) ranging from 0.09 to 0.48 μM.

#### 2.2.4. Thiol-Yne Addition

**Scheme 33:**
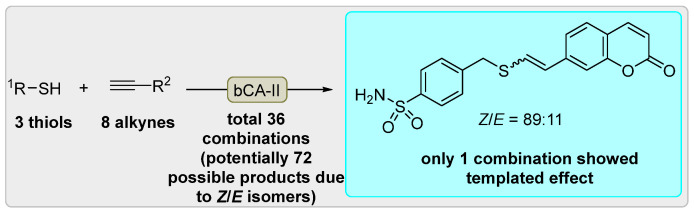
Binary fragment KTGS using bCA-II as the biological target.

The bCAII-templated thiol-yne ligation was effectively performed using a binary fragment KTGS approach, as demonstrated (Scheme 33) [122]. Out of the 36 possible combinations, only one was successfully templated by CAII, as confirmed by LC-MS/MRM and LC-HRMS analyses. This reaction produced a mixture of diastereoisomers with a *Z*/*E* ratio of 89:11, yielding an anti-Markovnikov thiol-yne product with no detectable traces of the Markovnikov thiol-yne product. Pure isomeric forms of the vinyl sulfide derivative (*Z*- and *E*-isomers) were chemically synthesized. The inhibitory activity of this isomerically pure vinyl sulfide derivative was then evaluated through 4-nitrophenyl acetate hydrolysis assays, indicating effective activity with IC_50_ values of 0.33 and 0.55 µM, respectively.

#### 2.2.5. Photo Reaction of Diazirine with Thiol

**Scheme 34:**
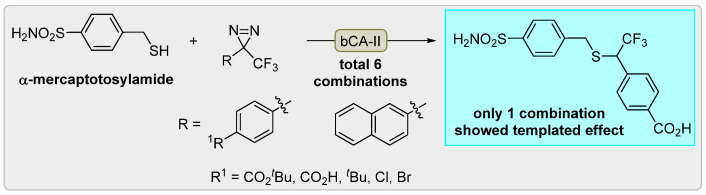
Binary fragment KTGS with bCA-II as the biological target via photo reaction.

Sabot et al. present an innovative method that integrates photochemistry with KTGS screening to identify inhibitors of CAII (Scheme 34) [123]. In their study, they investigated a library of diversely substituted aryl trifluoromethyl diazirines in a one-to-one binary mixture with α-mercaptotosylamide, which serves as an anchor molecule by forming a complex with the catalytic Zn^2+^ ion. The reaction mixtures were subjected to photo-irradiation at room temperature and subsequently analyzed using LC/MS-SIM. Only one was effectively templated by bCAII among the six possible combinations tested. Notably, the formation of product was significantly enhanced in the presence of bCAII compared to control experiments conducted without any enzyme or those including both enzymes and the commercial inhibitor, ethoxzolamide. The compound identified through this photochemical KTGS method exhibited an IC_50_ value of 307 nM, indicating a slight increase in activity compared to the anchor fragment, α-mercaptotosylamide, which has an IC_50_ of 360 nM.

### 2.3. C-C Bond Formation

The carbon–carbon (C-C) bond-forming reaction, such as the Knoevenagel condensation, has been effectively employed as a click reaction in KTGS for the identification of bivalent inhibitors. However, the literature reveals that relatively few C-C bond-forming reactions have been utilized within KTGS to date.

#### Knoevenagel Condensation

**Scheme 35:**
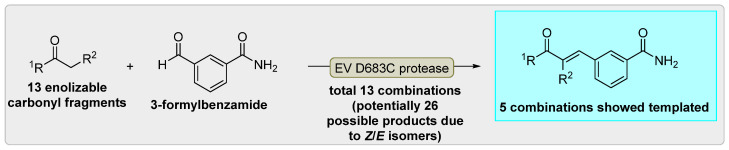
EV D683C protease-templated binary fragments KTGS screening.

Recent research by Rademann and co-workers has unveiled novel inhibitors targeting the enterovirus 3C-protease (EV protease) (Scheme 35) [124]. These inhibitors were identified through a Knoevenagel condensation binary fragment KTGS screening method, as illustrated in Scheme 35. Among the compounds analyzed, 3-formylbenzamide emerged as a moderately effective inhibitor of rhinovirus 3C-protease. A series of 13 enolizable carbonyl fragments were condensed with 3-formylbenzamide, both in the presence of and absent from the target protein, followed by analysis using HPLC-QTOF-MS. The findings revealed five notable hits from the KTGS screening that exhibited a remarkable increase in potency, up to 100 times more effective than 3-formyl benzamide, with IC_50_ values ranging from 0.46 to 2 µM. This advancement could significantly aid in developing therapeutic agents to combat these viral infections.

### 2.4. C-C and C-N Bond Formation

In recent years, multi-component click reactions that facilitate the simultaneous formation of carbon–carbon (C-C) and carbon–nitrogen (C-N) bonds have been utilized in the field of KTGS. Notably, two specific multi-component reactions—the Mannich reaction and the Ugi reaction—have proven effective in identifying inhibitors of the target within this context.

#### 2.4.1. Mannich Reaction

**Scheme 36:**
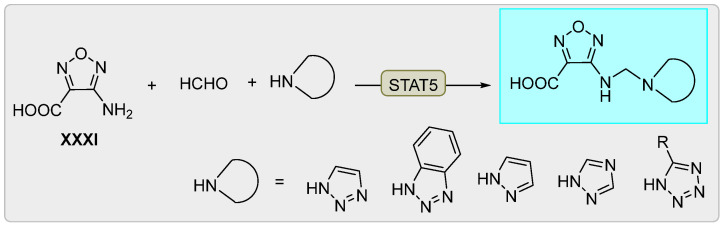
STAT5-assisted Mannich ligation.

Rademann et al. [125] identified inhibitors of the transcription factor STAT5 utilizing Mannich ligation (Scheme 36). The lead compound, 4-amino-furazan-3-carboxylic acid (**XXXI**), exhibited an inhibitory constant (*K_i_*) of 420 μM and was characterized as a STAT5 inhibitor through a fluorescence polarization (FP) assay. The fragment was reacted with formaldehyde and various *N*-heteroaryl nucleophiles, both in the presence and absence of STAT5 protein. Product formation occurred only in the presence of the target protein, suggesting a protein-dependent ligation reaction. For in situ Mannich ligation, **XXXI** was incubated with formaldehyde and one *N*-heteroaryl nucleophile per well in a microtiter plate. Incorporating 5-membered *N*-heterocycles into the formation of **XXXI** significantly enhanced STAT5 inhibition. Among the products, the tetrazol-1-yl substituted compound demonstrated the most potent inhibitory activity, with *K_i_* values ranging from 3.4 to 0.8 μM. This was followed by the 1,2,4-triazol-1-yl (*K_i_* = 48 μM), pyrazol-1-yl (*K_i_* = 122 μM), and 1,2,3-triazol-1-yl (*K_i_* = 191 and 189 μM) compounds. Active compounds were subsequently re-synthesized and evaluated in the FP assay. The inhibitor discovered through Mannich ligation was demonstrated to block the phosphorylation of the STAT5 protein in a cellular model of acute myeloid leukemia. Additionally, it inhibited the DNA-binding activity of STAT5 dimers and reduced the proliferation of cancer cells in a mouse model.

#### 2.4.2. Ugi Reaction

**Scheme 37:**
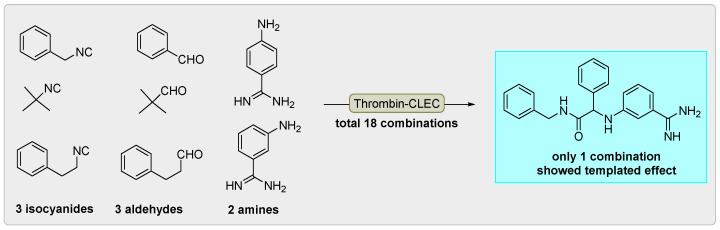
Multi-fragment KTGS via thrombin-CLEC-templated 3-component Ugi reaction.

Weber reported the discovery of a highly potent thrombin inhibitor achieved through implementing a three-component Ugi reaction, utilizing a multi-component KTGS strategy (Scheme 37) [126]. Notably, product formation was not observed in the absence of thrombin-CLEC. In contrast, the presence of thrombin-CLEC significantly enhanced product formation, which was subsequently confirmed by MALDI-TOF mass spectrometry. Of the eighteen potential combinations tested, only one displayed the templated effect. The resulting product exhibited substantial inhibitory activity, with an inhibition constant (*K_i_*) of 50 nM.

**Scheme 38:**
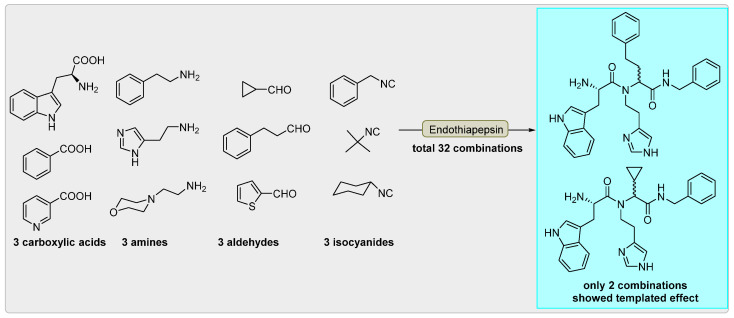
Multi-fragment KTGS using endothiapepsin-assisted 4-component Ugi reaction.

Hirsh’s research team investigated a four-component Ugi reaction, which was templated by endothiapepsin, employing a multi-fragment KTGS strategy (Scheme 38) [127]. Among the 32 potential products from the four-component Ugi reaction, their comprehensive and systematic analytical approach, utilizing UPLC-TQD-SIR, identified two promising candidates. These compounds demonstrated notable inhibitory potency, with IC_50_ values ranging from 1.3 to 3.5 µM, highlighting their potential significance in therapeutic applications.

## 3. Conclusions and Future Directions

Kinetic target-guided synthesis (KTGS) is an innovative strategy that significantly accelerates fragment-based drug discovery by offering a fundamentally new perspective on the identification and development of therapeutic agents. KTGS has demonstrated impressive efficacy across a broad range of biological targets, including enzymes, RNA, protein–protein interaction interfaces, and phosphate-binding sites, primarily in vitro. More recently, emerging studies have reported promising cellular applications of KTGS, underscoring its potential relevance in complex biological environments.

Beyond 1,3-dipolar cycloaddition, a range of carbon–nitrogen (C–N), carbon–sulfur (C–S), carbon–carbon (C–C), and dual carbon–nitrogen/carbon–carbon (C–N/C–C) bond-forming click reactions have been successfully applied in KTGS, leading to the discovery of novel inhibitors targeting diverse biological functions. Nevertheless, many potentially suitable click reactions remain largely unexplored within the KTGS framework. The development and incorporation of new reaction modalities hold considerable promise for expanding the scope and applicability of KTGS, thereby driving further innovation and advancement in this rapidly evolving field.

## Figures and Tables

**Table 1:** Click reactions reported in KTGS.

Sr. No.	Type of Click Reactions
1	C-N Bond Forming Reactions (a) Alkyne-Azide Huisgen Cycloaddition (1,3-Dipolar Cycloaddition) 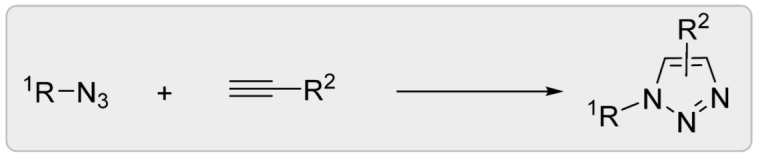 (b) Sulfo- or Seleno-Click Amidation 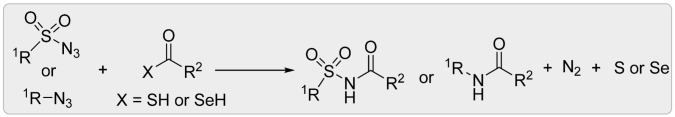 (c) Conventional Amidation 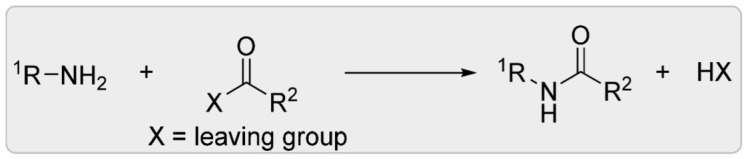 (d) Oxime Ligation 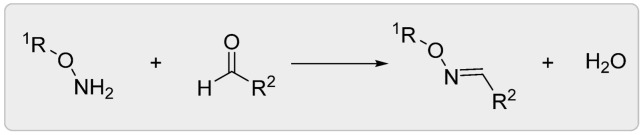 (e) *N*-Alkylation 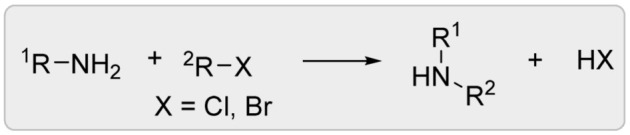
2	C-S Bond Forming Reactions (a) *S*-Alkylation 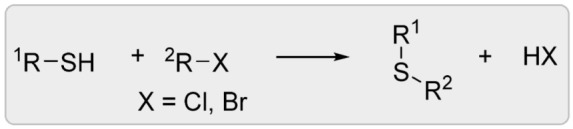 (b) Epoxide Ring Opening by Thiol 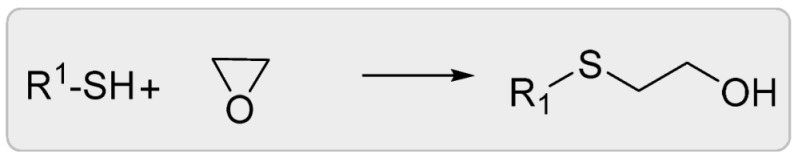 (c) Thio-Michael Addition 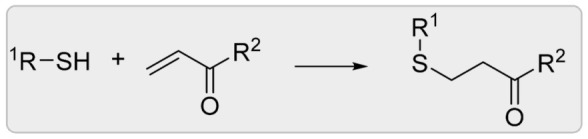 (d) Thiolyne Addition 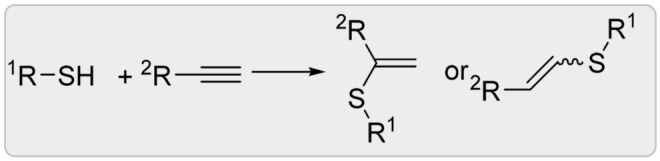 (e) Photo Reaction of diazirine with Thiol 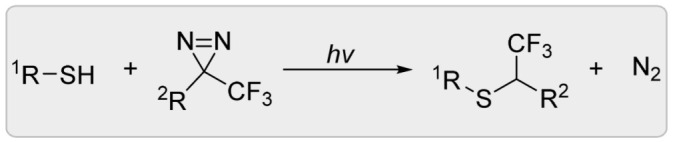
3	C-C Bond Forming Reactions (a) Knoevenagel Condensation 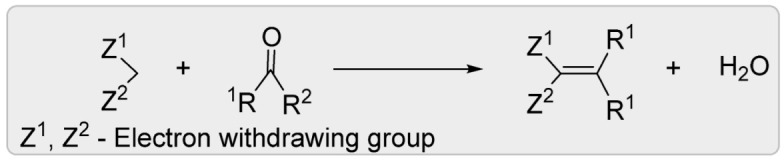
4	C-C/C-N Bond Forming Reactions (a) Mannich Reaction 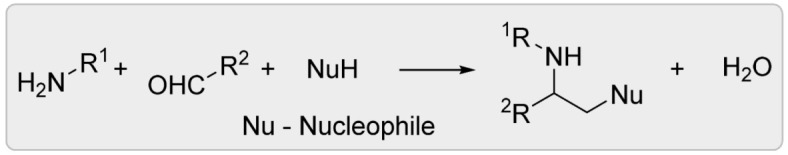 (b) Ugi Reaction 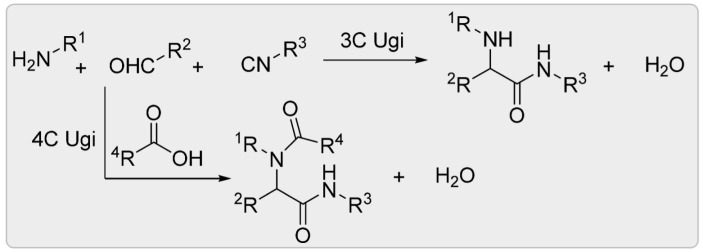

**Table 2:** Biological targets used and the type of click reactions performed for the identification of inhibitors via KTGS.

Sr. No.	Biological Targets Used in KTGS and Their General Function	Type of Click Reactions Performed with the Target
1	Acetylcholinesterase (AChE) AChE is crucial for the hydrolysis of acetylcholine and other neurotransmitters within both the central and peripheral nervous systems [17,18]. Its inhibition is a therapeutic strategy employed in the management of Alzheimer’s disease, where AChE inhibitors serve to enhance cholinergic signaling and mitigate cognitive decline associated with the condition [19].	1,3-Dipolar cycloaddition, Thio-Michael addition
2	Acetylcholine binding proteins (AChBPs) Nicotinic acetylcholine receptors (nAChRs) are part of a larger superfamily of neurotransmitter ligand-gated ion channels. These receptors are recognized as promising therapeutic targets for various central nervous system disorders, including schizophrenia, nicotine dependence, and Alzheimer’s disease [20,21,22]. The acetylcholine-binding proteins (AChBPs) share structural homology with the extracellular domains of pentameric ligand-gated ion channels, providing valuable insights into the receptor’s function and pharmacology.	1,3-Dipolar cycloaddition
3	Carbonic anhydrase II (CAII) CAII is a zinc-dependent metalloenzyme that plays crucial roles in several physiological processes, including cellular respiration and the regulation of CO_2_ and bicarbonate transport. They are also pivotal in acid-base homeostasis, bone resorption, and modulation of calcification. Additionally, CAII has been implicated in tumorigenesis, underscoring its importance in both normal physiological function and pathological states [23].	1,3-Dipolar cycloaddition, *S*-Alkylation, Thiol-yne addition, Photo reaction
4	Ethionamide repressor (EthR) EthR is a transcriptional regulator in mycobacteria that significantly influences the sensitivity of *Mycobacterium tuberculosis* to various antibiotics. When EthR is genetically inactivated, its levels increase, thereby enhancing the susceptibility of mycobacterial strains to the antibiotic ethionamide [24].	1,3-Dipolar cycloaddition
5	Abelson (ABL) The ABL family of tyrosine kinases, comprising ABL1 and ABL2, plays a critical role in regulating cellular processes essential for development and maintaining normal physiological conditions. However, during tumor progression, metastasis, tissue injury responses, inflammation, and neural degeneration, these ABL kinases can become inappropriately activated, contributing to the progression of various diseases [25].	1,3-Dipolar cycloaddition
6	Ribosome Ribosomes serve as the central components of the translation machinery in all organisms. The translation represents a crucial step in gene expression, as it converts the genetic information encoded in messenger RNAs (mRNAs) into continuous chains of amino acids (polypeptides or proteins) that possess structural and/or catalytic functions. Ribosomes perform the following two primary roles: decoding the genetic message and facilitating the formation of peptide bonds [26].	1,3-Dipolar cycloaddition
7	Botulinium neurotoxin serotype A (BoNT/A) BoNT/A is a zinc-dependent protease known for inhibiting the acetylcholine in response to calcium (Ca^2+^) signals. The structure of BoNT/A comprises the following two key components: a heavy chain that binds receptors and a light chain that harbors catalytic activity, linked by a disulfide bond. This mechanism enables BoNT/A to disrupt neurotransmission effectively [10].	1,3-Dipolar cycloaddition
8	D-amino acid oxidase (DAO) DAO is essential for catalyzing the oxidative deamination of D-amino acids, producing the corresponding α-keto acids, ammonia, and hydrogen peroxide. In humans, DAO is especially significant for its ability to convert D-serine, a neuromodulator involved in synaptic transmission and plasticity, into hydroxypyruvate via deamination. Proper regulation of D-serine levels by DAO is critical, as imbalances can have significant effects on neurological function and have been implicated in disorders such as schizophrenia and amyotrophic lateral sclerosis [27].	1,3-Dipolar cycloaddition
9	Biotin protein ligase (BPL) BPL is an essential enzyme found in all organisms, playing a crucial role in the post-translational attachment of biotin to a specific lysine residue located in the active site of biotin-dependent enzymes. Inhibitors targeting this vital metabolic enzyme, BPL, offer a promising avenue for developing new antibacterial drugs [28].	1,3-Dipolar cycloaddition
10	Aspartic protease endothiapsin Aspartic proteases constitute a diverse family of proteolytic enzymes distinguished by the presence of two highly conserved aspartic acid residues within their active sites, which are indispensable for catalytic activity. These enzymes are ubiquitously distributed across a broad spectrum of biological taxa, including fungi, vertebrates, plants, and retroviruses such as HIV. Their functional versatility allows them to participate in essential physiological and pathological processes, and their activity is closely linked to the development and progression of diseases such as hypertension, malaria, Alzheimer’s disease, and AIDS [29]. Endothiapepsin, a well-characterized fungal aspartic protease, is frequently employed as a structural and functional model in drug discovery owing to its significant homology with clinically relevant aspartic proteases.	1,3-Dipolar cycloaddition, Ugi reaction
11	Chitinase Chitin is a constituent of fungal cell walls, the exoskeletons of crustaceans and insects, and the microfilarial sheaths of parasitic nematodes [30,31,32]. Chitinases have emerged as promising molecular targets for the development of antifungal, insecticidal, and antiparasitic agents, owing to their pivotal role in chitin degradation [33]. In addition to their significance in pathogen control, chitinases have garnered attention for their therapeutic potential in human medicine, particularly in the context of asthma and other chitin-associated inflammatory disorders [34].	1,3-Dipolar cycloaddition
12	Insulin-degrading enzyme (IDE) IDE is a zinc metalloprotease belonging to the M16 family, characterized by its broad substrate specificity and pivotal role in the catabolism of several physiologically significant peptides. IDE exhibits proteolytic activity towards insulin [35], amyloid-β [36], insulin-like growth factor II (IGF-II) [37], glucagon [38], amylin [39], and somatostatin [40], thereby regulating their extracellular and intracellular concentrations. This enzymatic activity emphasizes IDE’s significance in various metabolic processes and its potential implications in conditions such as diabetes and Alzheimer’s disease.	1,3-Dipolar cycloaddition
13	Cyclooxygenase-2 (COX-2) Cyclooxygenase (COX) enzymes constitute a critical class of heme-containing isozymes that mediate the oxygenation of arachidonic acid to yield prostanoids. Dysregulation of COX activity has been implicated in the pathogenesis of a variety of pathological conditions, including inflammatory diseases, cardiovascular disorders, and certain cancers [41,42,43,44,45]. Consequently, COX enzymes represent key molecular targets for the development of anti-inflammatory and analgesic pharmacological agents.	1,3-Dipolar cycloaddition
14	HIV-1 protease (HIV-1-Pr) HIV-1-Pr is a critical enzyme unique to the HIV-1 virus. It plays an essential role in the virus’s maturation by cleaving precursor polyproteins, specifically gag and gag-pol. This processing is vital for the correct assembly and replication of the retrovirus, which positions HIV-1-Pr as a key target for therapeutic strategies in the treatment of HIV/AIDS [46].	1,3-Dipolar cycloaddition
15	B-cell lymphoma-extra-large (Bcl-xL) Bcl-xL, a key member of the Bcl-2 protein family, is vital in regulating the intrinsic apoptosis pathway, the programmed cell death mechanism essential for maintaining cellular homeostasis and tissue development. Bcl-xL primarily acts as an anti-apoptotic factor by inhibiting the activation of pro-apoptotic proteins such as Bak and Bax. The balance between Bcl-xL and the pro-apoptotic members of the Bcl-2 family is crucial for determining cell survival and death. Dysregulation of Bcl-xL expression has been linked to the pathogenesis of various cancers and other diseases [47].	Sulfo-click amidation
16	Myeloid cell leukemia 1 (Mcl-1) Mcl-1 is a multifaceted regulatory protein primarily recognized for its anti-apoptotic function within the Bcl-2 family. The Mcl-1 protein plays a crucial role in safeguarding cells from apoptosis under various conditions that induce cell death. Research has demonstrated that Mcl-1 is a vital pro-survival factor across multiple tumor types, leading to the recent development of Mcl-1-specific BH3-mimetics currently being evaluated in clinical trials [48].	Sulfo-click amidation, Seleno-click amidation
17	Factor Xa Protein factor Xa is an essential serine protease in blood coagulation. It plays a crucial role in activating the coagulation cascade, making it a significant target for various antithrombotic medications to prevent blood clots [49].	Conventional amidation
18	Glycinamide ribonucleotide transformylase (GAR TFase) GAR TFase is a folate-dependent enzyme that catalyzes a critical step in the de novo purine biosynthesis pathway, specifically mediating the transfer of a formyl group from 10-formyltetrahydrofolate to glycinamide ribonucleotide (GAR) to produce formylglycinamide ribonucleotide (FGAR). This reaction is essential for the generation of purine nucleotides, which are fundamental to nucleic acid synthesis and cellular proliferation. Due to its indispensable role in nucleotide biosynthesis, GAR TFase has long been recognized as a strategic molecular target for antineoplastic agents [50]. The primary role of GAR TFase is to formylate GAR.	*N*-Alkylation
19	14-3-3 The 14-3-3 protein family comprises highly conserved, ubiquitously expressed dimeric proteins that function as key regulators of numerous intracellular signaling pathways, principally those mediated by serine/threonine kinases. The involvement of 14-3-3 proteins in these pathways underscores their role in maintaining cellular homeostasis and responding to diverse physiological stimuli. Their dysregulation has been implicated in several diseases, including cancer and neurodegenerative disorders, making them significant targets for therapeutic intervention [51].	Epoxide ring formation Oxime ligation
20	Thrombin Thrombin is a member of the serine protease family, which converts fibrinogen into fibrin (an integral step in clot formation). Thrombin also participates in various other functions, including the initiation of inflammation, neoplastic transformation, angiogenesis, atherosclerosis, and tissue repair [52].	Ugi reaction
21	Transcription factor STAT5 STAT5 is a transcription factor that becomes constitutively activated in various malignancies, where it regulates the expression of genes involved in cell proliferation, survival, and tumor progression [53]. Dysregulation or sustained activation of STAT5 has been linked to several malignancies, underscoring its potential as a therapeutic target.	Mannich reaction
22	Cellular Src kinase (c-Src) c-Src is an essential non-receptor tyrosine kinase that regulates various physiological processes and oncogenic activities. It is key to cellular signaling pathways, affecting normal cellular functions, and is associated with multiple cancer-related mechanisms. Its involvement in benign and malignant transformations underscores c-Src’s significance within the complex landscape of cell biology and cancer research [54].	Thio-Michael addition
23	Enterovirus 3C-protease (EV protease) The Enterovirus 3C protease (EV protease) is crucial to the intricate dynamics of viral immune evasion. It is a well-established target for therapeutic interventions to treat enteroviral and rhinoviral infections. Studying the functions and mechanisms of the Enterovirus 3C protease provides valuable insights that can help develop effective strategies to combat viral infections [55].	Knoevenagel condensation
24	Endoplasmic Reticulum Aminopeptidase 2 (ERAP2) ERAP2 is a zinc-dependent metallopeptidase localized within the lumen of the endoplasmic reticulum, where it plays a crucial role in antigen processing for presentation by the Class-I major histocompatibility complex. This process is essential for initiating immune responses against infected cells. Variations in the ERAP2 gene, particularly single-nucleotide polymorphisms, have been linked to increased susceptibility to chronic inflammatory disorders, such as Crohn’s disease, while also providing some protection against severe infections, such as pneumonia [56].	1,3-Dipolar cycloaddition

## Data Availability

No new data were created or analyzed in this study. Data sharing is not applicable to this article.

## Abbreviations

The following abbreviations are used in this manuscript:

TGS	Target-guided synthesis
DCC	Dynamic combinatorial chemistry
KTGS	Kinetic target-guided synthesis
AChE	Acetylcholinesterase
AChBPs	Acetylcholine binding proteins
nAChRs	Acetylcholine binding proteins
CAII	Carbonic anhydrase II
EthR	Ethionamide repressor
ABL	*Abelson*
BoNT/A	Botulinum neurotoxin serotype A
DAO	D-amino acid oxidase
BPL	Biotin protein ligase
IDE	Insulin-degrading enzyme
COX-2	Cyclooxygenase-2
HIV-1-Pr	HIV-1 protease
Bcl-xL	B-cell lymphoma-extra-large
Mcl-1	Myeloid cell leukemia 1
GAR TFase	Glycinamide ribonucleotide transformylase
FGAR	Formylglycinamide ribonucleotide
c-Src	Cellular Src kinase
EV protease	Enterovirus 3C-protease
ERAP2	Endoplasmic reticulum aminopeptidase 2
BSA	Bovine serum albumin
SmChi	Serratia marcescens chitinases
ITT	Insulin tolerance test
Fm	9-Fluorenylmethyl
Glck	Glucokinase
pFLA	Pathogenic free-living amoebae
MAI	Multi-substrate adduct inhibitor
